# Comparative Effects of Drying Methods on Waste Seed Melon Pulp By-Product Quality and Polysaccharide Characteristics: Structural Features, Bioactivity, and Gastrointestinal Digestion Stability

**DOI:** 10.3390/foods15193445

**Published:** 2026-09-27

**Authors:** Jingjing Sun, Mingxi Jia, Fei Yang, Zhen Wang, Yue Cui, Xiuli Yang, Nige’er’reyi Yadika’er, Haixia Han

**Affiliations:** 1 College of Food Science and Pharmacy, Xinjiang Agricultural University, Urumqi 830052, China; 320242546@stu.xjau.edu.cn (J.S.); 120240097@xjau.edu.cn (M.J.); 15609973796@163.com (Z.W.); 320242506@stu.xjau.edu.cn (Y.C.); 320242565@stu.xjau.edu.cn (X.Y.); nigary-sy@xjau.edu.cn (N.Y.); 2 Xinjiang Key Laboratory of Postharvest Fruit Science and Technology, Urumqi 830052, China; 3 College of Modern Industry in Chinese Herbal Medicine, Xinjiang Agricultural University, Urumqi 830052, China; 4 Xinjiang Uygur Autonomous Region Science and Technology Resource Sharing Service Center, Urumqi 830052, China; yangfei4314@sina.com

**Keywords:** *Citrullus lanatus* var. *megalospermus*, drying methods, structural characterization, antioxidant activity, structure–activity relationship

## Abstract

Waste seed melon processing by-products are rich in bioactive polysaccharides. Nevertheless, their high moisture content and perishability restrict the high-value utilization of these polysaccharides. In this study, four pretreatment drying methods, namely sun drying (SD), 55 °C hot-air drying (HAD), weak microwave-assisted hot-air drying (WMHAD), and vacuum freeze drying (VFD), were adopted to investigate the effects of drying methods on the quality characteristics of dried seed melon, as well as the structural characteristics (micromorphology, monosaccharide composition, molecular weight distribution, and functional groups), biological activities (antioxidant capacity, α-glucosidase inhibitory activity, and pancreatic lipase inhibitory activity), and gastrointestinal stability of seed melon polysaccharides (SMPs). The structure–activity relationship of SMPs was further elucidated. The results showed that none of the four drying methods disrupted the glycosidic backbone of polysaccharides, only altering the molar ratios of monosaccharides. VFD preserved the loose structure of pulp; VFD-SMP possessed the highest weight-average molecular weight and galacturonic acid content and exhibited optimal α-glucosidase inhibitory activity and gastrointestinal stability. HAD induced polysaccharide degradation, and HAD-SMP displayed the strongest antioxidant capacity. WMHAD-SMP exerted prominent pancreatic lipase inhibitory activity. The SD-treated samples exhibited the lowest polysaccharide extraction yield as well as the poorest overall biological activities. This work may provide a preliminary theoretical basis and data reference for the targeted preparation of functional polysaccharides from seed melon processing by-products.

## 1. Introduction

Seed melon (*Citrullus lanatus* var. *megalospermus*) adapts to arid environments with substantial diurnal temperature differences. As an economic crop, it has been extensively cultivated on a large scale in numerous countries worldwide and delivers prominent industrial economic benefits. Similar to other Cucurbitaceae crops, seed melon exhibits considerable morphological and quality diversity [1]. Its processing by-products, especially the pulp, possess great potential for the development of functional ingredients. However, the current seed melon processing industry prioritizes seeds and discards most of the pulp by-products, resulting in severe resource waste and restricting the high-value development of the seed melon industrial chain. Studies have demonstrated that polysaccharides derived from fruit and vegetable by-products exhibit low toxicity, strong antioxidant capacity, glycolipid metabolism regulation and various other bioactivities, showing broad application prospects in functional foods, natural food additives and dietary intervention fields [2]. Nevertheless, fresh pulp has a high moisture content and vigorous respiration, rendering it susceptible to postharvest spoilage and making long-term storage and long-distance transportation difficult. Drying pretreatment is an indispensable strategy for preserving raw material quality and facilitating the efficient utilization of active components [3]. Although emerging technologies such as nanotechnology have shown promise in enhancing fruit crop quality and yield [4], conventional drying remains the most practical and widely adopted pretreatment for perishable by-products. Different drying methods vary substantially in their heat and mass transfer mechanisms, processing temperature, oxygen atmosphere, and dehydration rate, all of which affect the microstructure of fruit and vegetable tissues, the retention of active components, and functional performance. For instance, Hao [5] investigated the drying of kiwifruit and confirmed that freeze drying generates a uniform and loose porous structure, preserving cellular integrity to the greatest extent; hot-air drying at 60 °C induces remarkable tissue shrinkage and pore collapse, leading to dense and rough sample surfaces. Zielinska [6] reported that polyphenol contents declined under all hot-air drying treatments. The contents of polyphenols and anthocyanins under single microwave vacuum drying at 1.3 W/g were lower than those of hot-air-dried samples, whereas combined hot-air–microwave drying at 90 °C achieved optimal retention of polyphenols and anthocyanins as well as superior antioxidant capacity. Li [7] explored the drying characteristics of two types of apple pomace and found that concentrated apple pomace treated by industrial drum drying exhibited significantly stronger DPPH, ABTS and FRAP antioxidant activities compared with samples subjected to hot-air drying and heat pump drying. Freeze-dried samples possessed the highest water-holding and oil-holding capacities. Apple pomace processed by hot-air drying and heat pump drying suffered severe polyphenol loss, accompanied by the weakest antioxidant capacity and inferior functional properties. Different drying methods exert remarkably disparate regulatory effects on the quality of fruit and vegetable polysaccharides, especially when comparing hot-air drying and vacuum freeze drying, which yield distinctly divergent outcomes. High-temperature hot-air drying induces the cleavage of polysaccharide glycosidic bonds and thermal degradation of macromolecular chains. Meanwhile, Maillard browning products can adsorb onto polysaccharide fractions, reducing polysaccharide purity and darkening sample color. In contrast, vacuum freeze drying may help avoid the aforementioned drawbacks and better preserve the structural integrity and antioxidant activity of polysaccharides to the maximum extent [8,9]. Numerous studies have verified such discrepancies. In the research on Schizophyllum commune polysaccharides conducted by Fan [10], the total sugar content of freeze-dried polysaccharides (46.07 ± 0.08%) was higher than that of polysaccharides obtained via hot-air drying (45.31 ± 0.34%), heat pump drying (42.61 ± 0.30%) and vacuum drying (43.09 ± 0.48%). Low-temperature freeze drying may help prevent heat-induced thermal degradation of polysaccharides and Maillard reactions, and better retain polysaccharide components, which explains why freeze-dried polysaccharides generally possess higher total sugar contents than those subjected to thermal drying. Shang [11] reported that lithospermum root polysaccharides prepared by freeze drying exhibited significantly stronger DPPH and ABTS radical scavenging capacities as well as reducing power compared with samples treated by hot-air drying and vacuum drying. Freeze drying is also favorable for maintaining desirable physicochemical properties and antioxidant activities of polysaccharides. Wang [12] further confirmed that the high-temperature environment during hot-air drying may trigger molecular aggregation and chain degradation of Angelica sinensis polysaccharides, thereby diminishing the bioactivities of polysaccharides.

Furthermore, different drying pretreatments exert varying degrees of effects on the gastrointestinal stability of dried fruits and vegetables, thereby altering their biological activities [13]. It is worth noting that most existing evaluations of polysaccharide bioactivities are confined to in vitro buffer systems, while neglecting the regulatory effect of gastrointestinal digestive degradation on the actual functional performance of polysaccharides. After undergoing strong acid hydrolysis in the stomach and pancreatic enzymatic hydrolysis in the small intestine, polysaccharides experience molecular weight degradation, structural reconstruction, and functional activity remodeling, which further lead to differential changes in biological activities [14]. To date, there have been no systematic studies on waste seed melon pulp by-products that synchronously compare four drying techniques, including sun drying (SD), 55 °C hot-air drying (HAD), weak microwave-assisted hot-air drying (WMHAD), and vacuum freeze drying (VFD), and comprehensively correlate the quality of dried products, the multi-level structure of seed melon polysaccharides (SMPs), multi-target bioactivities, and gastrointestinal digestive stability. Accordingly, comparing the effects of diverse drying methods on the quality of dried seed melon products and the bioactivity of their polysaccharides is of great theoretical and practical significance for the intensive processing and high-value utilization of seed melon resources.

Based on the above research gaps, this study took waste seed melon pulp by-products from Xinjiang as the research material. Four common drying methods, namely SD, HAD, WMHAD and VFD, were adopted. The effects of different drying methods on the physicochemical quality of dried seed melon and polysaccharide extraction efficiency were systematically analyzed. The structural differences of SMPs were further elucidated from the perspectives of monosaccharide composition, molecular weight distribution, micromorphology, and infrared functional groups. Combined with the evaluation of in vitro antioxidant capacities, α-glucosidase and pancreatic lipase inhibitory activities, and in vitro simulated gastrointestinal digestion characteristics, the present work explored the structure–activity relationship among drying-induced polysaccharide structural evolution, functional bioactivities, and digestive stability. Meanwhile, the potential application scenarios of the four drying processes were preliminarily discussed. This study aims to provide theoretical support for the resource utilization and high-value exploitation of seed melon by-products and the targeted preparation of functional polysaccharides.

## 2. Materials and Methods

### 2.1. Materials

Half-ripened large-seed seed melon fruits (black seed cultivar) were harvested in August 2025 in Fukang City, Changji Hui Autonomous Prefecture, Xinjiang Uygur Autonomous Region, China. In the seed melon industry, seeds serve as the primary product, while pulp is generally discarded as waste. To guarantee sample freshness and uniform maturity, intact fruits were collected, washed, and manually separated to remove the seeds. The resulting pulp, regarded as a waste seed melon pulp by-product, was refrigerated at 4 ± 0.5 °C and processed within 24 h after harvest.

Cellulase (400 U/mg), α-glucosidase (50 U/mg), and p-nitrophenyl-α-D-glucopyranoside (PNPG) were purchased from Yuanye Bio-Technology Co., Ltd. (Shanghai, China). Pancreatic lipase (15–35 U/mg), orlistat, and 4-nitrophenyl butyrate (PNPB) were obtained from Aladdin Biochemical Technology Co., Ltd. (Shanghai, China). Galacturonic acid and its standard substance, together with 2,2-diphenyl-1-picrylhydrazyl (DPPH, purity ≥ 98.5%) and 2,2′-azino-bis (3-ethylbenzothiazoline-6-sulfonic acid) diammonium salt (ABTS, purity ≥ 99%), were supplied by Macklin Biochemical Co., Ltd. (Shanghai, China). All conventional chemical reagents, including absolute ethanol, phenol, sulfuric acid, sodium carbonate, and trichloroacetic acid, were of analytical grade and purchased from Sinopharm Chemical Reagent Co., Ltd. (Shanghai, China).

### 2.2. Preparation of Dried Seed Melon Products with Different Drying Methods

Waste seed melon pulp by-products free of diseases, insect pests, rot and deterioration were selected, thoroughly cleaned, and uniformly cut into 0.5 cm thick slices. The slices were dried to constant weight via four distinct drying techniques within a total drying duration of 72 h. In this study, constant weight was defined as a mass change of ≤0.1 g or ≤0.5% between two consecutive weighings at 2 h intervals (for WMHAD, every five cycles), with no further decrease in mass. The standardized operation procedures and quality control protocols for each drying method are described as follows.

SD: Seed melon slices were spread in a single layer on drying nets and naturally dehydrated under outdoor-ventilated and sunny conditions. To ensure uniform drying, the samples were turned over every 2 h during daytime. At night, the samples were transferred indoors and sealed to prevent mold growth caused by dew infiltration. All samples were dried to constant weight within a total drying period of 72 h. Moldy and abnormal samples were eliminated during the experiment.

HAD: Samples were arranged in a single layer inside a thermostatic drying oven (DHG-9240A, Shanghai Shenxian Thermostatic Equipment Factory, Shanghai, China) and dehydrated by natural convective hot air at a constant temperature of 55 °C for 24 h until constant weight was reached.

WMHAD: Samples were evenly spread on a sample tray lined with two layers of cotton gauze cloth and positioned in the central zone of a weak microwave-assisted hot-air drying apparatus (ORW2S-5G, Nanjing Orient Microwave Technology Co., Ltd., Nanjing, China). Drying parameters were set as follows: The total microwave power was maintained at 1000 W, with three emitters activated simultaneously. Hot-air circulation was regulated at a temperature of 30 °C, an air velocity of 4 m/s, and a relative humidity of 29%. The drying process consisted of alternating cycles of microwave irradiation (150 s) and hot-air exposure (8 min), and the samples were dried for 10 h, with the sample weight recorded every five cycles until constant weight was achieved.

VFD: Samples were pre-frozen at −20 °C for 24 h and then transferred into a vacuum freeze dryer (Scientz-10N/C, Ningbo Scientz Biotechnology Co., Ltd., Ningbo, China), where they were freeze-dried for 48 h under a cold trap temperature of −50 °C and a vacuum pressure below 10 Pa until constant weight was achieved.

### 2.3. Visual Photographs and Scanning Electron Microscopy (SEM) Observation of Dried Seed Melon

Macroscopic photographs of dried seed melon samples prepared by the four drying methods were captured. Representative slices of dried seed melon were cut, fixed on specimen stubs with conductive adhesive, and subjected to vacuum gold sputtering to improve the electrical conductivity of samples. The surface and cross-sectional microstructure of dried seed melon was observed by scanning electron microscopy at an accelerating voltage of 2.0 kV. SEM micrographs of the surface and cross-section were acquired at appropriate magnifications.

### 2.4. Moisture Content, Rehydration Ratio, and Color Measurements

(1) Moisture content

The moisture content of dried seed melon samples was determined using a halogen moisture analyzer (DHS-16, Jinghai Instrument Co., Ltd., Shanghai, China). Each sample was measured in triplicate, and the results were expressed as the mean value.

(2) Rehydration ratio

Dried seed melon samples were rehydrated in a water bath at 30 °C with a solid-to-liquid ratio of 1:100 (g:mL). After 4 h of rehydration, the weight of the samples was recorded. All determinations were performed in triplicate, and the results were presented as mean values. The rehydration ratio was calculated according to Equation (1):

R_f_ (%) = (m_t_ − m_0_)/m_0_ × 100 (1)

where: R_f_—rehydration ratio, %; m_0_—mass of the sample before rehydration, g; m_t_—mass of the sample after 4 h of rehydration, g.

(3) Color measurements

Color parameters were measured using a colorimeter (JZ-350, Jinzhun Instrument Equipment Co., Ltd., Shenzhen, China). The lightness value (L*; 0 = black; 100 = white), red–green coordinate (a*; positive value represents red; negative value represents green), and yellow–blue coordinate (b*; positive value represents yellow; negative value represents blue) of dried seed melon samples were determined. Each sample was tested in triplicate, and the results were expressed as mean values. The total color difference (ΔE) was calculated using Equation (2):

(2) $$\Delta E=\sqrt{{\left(L^{*}\;-\;L_{0}^{*}\right)}^{2}+{\left(a^{*}\;-\;a_{0}^{*}\right)}^{2}{+\left(b^{*}\;-\;b_{0}^{*}\right)}^{2}}$$

### 2.5. Extraction of SMPs

Dried seed melon samples treated with four different drying methods were pulverized and sieved through a 100-mesh sieve. The powder was defatted by immersion in petroleum ether at a solid-to-liquid ratio of 1:3 (g:mL) for 12 h to prepare defatted seed melon powder. Exactly 100 g of defatted seed melon powder was accurately weighed and mixed with distilled water at a solid-to-liquid ratio of 1:10 (g:mL). Subsequently, 1.5% (*w*/*w*, relative to the mass of defatted seed melon powder) cellulase was added to the mixture, followed by enzymatic hydrolysis in a thermostatted water bath at 55 °C for 2.5 h. After hydrolysis, ultrasonic-assisted extraction was performed for 30 min (55 °C, 70 W, 50 Hz). Thereafter, the enzymatic reaction was terminated by inactivation in a boiling-water bath for 10 min. The extracting solution was separated by suction filtration, and the filter residue was re-extracted once using the same procedure. The two batches of filtrates were combined and concentrated via rotary evaporation. Four volumes of absolute ethanol were added into the concentrated solution. After thorough mixing, the solution was kept stationary at 4 °C for 12 h for ethanol precipitation. The precipitate was collected and vacuum freeze-dried at −50 °C and <10 Pa to constant weight to obtain crude SMP. The yield of crude polysaccharides (calculated based on defatted seed melon powder, %) was calculated according to Equation (3):

Yield (%) = m/M × 100(3)

where: m—dry mass of crude polysaccharides, g; M—mass of defatted seed melon powder, g.

Crude SMPs were dissolved to prepare a solution of 10 mg/mL. Proteins were removed via the Sevage method, followed by vacuum concentration. The concentrate was dialyzed (MWCO 3500 Da) for 48 h to remove low-molecular-weight impurities, and the retentate was collected and freeze-dried to obtain SMPs. Polysaccharides obtained from seed melon subjected to the four different drying treatments were designated as SD-SMP, HAD-SMP, WMHAD-SMP, and VFD-SMP, respectively.

### 2.6. SMP Chemical Composition

The total carbohydrate content was determined by the phenol–sulfuric acid method using glucose as the standard [15]. The protein content was measured via the Coomassie brilliant blue assay with bovine serum albumin (BSA) as the standard [16]. The uronic acid content was quantified by the sulfuric acid–carbazole method using galacturonic acid as the standard [17]. All measurements were performed in triplicate.

### 2.7. SMP Structural Characteristics

(1) SEM

Appropriate amounts of SMP samples were evenly dispersed on conductive adhesive. After vacuum gold sputtering treatment, the samples were transferred into the SEM chamber. The surface morphology and microstructural characteristics were observed and recorded at an accelerating voltage of 2.0 kV under various magnifications.

(2) Monosaccharide composition

Briefly, 2.5 mg of polysaccharide sample was mixed with 1 mL of 2 M trifluoroacetic acid (TFA) solution and hydrolyzed at 120 °C for 2 h. The solvent was evaporated under a nitrogen stream. The residue was washed with 1 mL of methanol and dried again, and the methanol washing operation was repeated 2–3 times. Subsequently, the residue was dissolved in 1 mL of sterile water to prepare polysaccharide hydrolysate for subsequent use. Then, 0.2 mL of polysaccharide hydrolysate was mixed with 0.2 mL of 0.5 mol/L sodium hydroxide solution and 0.5 mL of 0.5 mol/L methanolic PMP solution. After vortex mixing, the mixture was derivatized in a water bath at 70 °C for 1 h. Upon completion of the reaction, 0.2 mL of 0.5 mol/L HCl solution was added for neutralization. Excess PMP was removed by triple extraction with 1 mL chloroform via vortexing. After discarding the chloroform layer, 0.3 mL of the aqueous phase was diluted with water to a final volume of 1 mL. The solution was filtered through a 0.45 μm organic microporous membrane prior to HPLC analysis. HPLC conditions: Analyses were performed on a Thermo U3000 liquid chromatography system (Dionex U3000, Thermo Fisher Scientific, Waltham, MA, USA) equipped with an Agilent ZORBAX Eclipse XDB-C18 column (4.6 mm × 250 mm, 5 μm, Agilent Technologies, Inc., Santa Clara, CA, USA). Isocratic elution was carried out using acetonitrile–phosphate buffer (12 g/L KH_2_PO_4_ solution, pH adjusted to 6.8 with 2 mol/L NaOH, volume ratio = 17:83) as the mobile phase. The flow rate was set at 0.8 mL/min, column temperature at 30 °C, detection wavelength at 250 nm, and injection volume at 10 μL. Rhamnose (Rha), arabinose (Ara), galactose (Gal), glucose (Glc), xylose (Xyl), mannose (Man), galacturonic acid (GalA), glucuronic acid (GlcA), glucosamine hydrochloride (GlcN), galactosamine hydrochloride (GalN), and fucose (Fuc) were used as monosaccharide standards. Monosaccharides were identified by retention time and quantified using the external standard method, and the molar percentage of each monosaccharide was calculated. All samples were analyzed in triplicate.

(3) Molecular weight distribution

Briefly, 5 mg of polysaccharide sample was dissolved in 1 mL of 0.05 M NaCl solution to prepare a sample solution at a concentration of 5 mg/mL. The solution was filtered through a 0.22 μm microporous membrane and transferred into a 2 mL sample vial for subsequent analysis. The molecular weight distribution was determined via high-performance gel permeation chromatography (HPGPC) using two serial-connected aqueous size-exclusion chromatography columns.

Instrumentation: Waters high-performance liquid chromatography system (Waters 1515, Waters Corporation, Milford, MA, USA); detector: Waters refractive index detector (Waters 2414, Waters Corporation, Milford, MA, USA); columns: two polymer-based aqueous SEC (GFC) columns (8 × 300 mm, Showa Denko K.K., Tokyo, Japan) connected in series; mobile phase: 0.05 M NaCl aqueous solution; flow rate: 0.50 mL/min; column temperature: 40 °C; injection volume: 30 μL. A series of carbohydrate standards with different molecular weights (180, 666, 6000, 9800, 21,900, 46,500, 110,000, 193,000, 353,000, 692,000 and 1,060,000) were accurately weighed and dissolved in 0.05 M NaCl solution to prepare serial standard solutions. All standard solutions were filtered through a 0.22 μm microporous membrane prior to analysis. Subsequently, HPGPC detection was performed using two connected-in-series high-performance gel permeation chromatography columns to establish the calibration curve. The molecular weight parameters of each polysaccharide sample were calculated based on the calibration curve of standard carbohydrates.

For SMPs exhibiting multiple HPGPC peaks, the overall weight-average molecular weight (overall *Mw*) was calculated from the individual peak *Mw* values and their relative peak areas. The calculation formula was as follows:

(4) $$Mw,\;overall\;(kDa)=\frac{\sum_{i}\left(A_{i}\times M_{w,i}\right)}{\sum_{i}A_{i}}$$

where $A_{i}\;$ is the relative peak area (%) of fraction i, and $M_{w,i}$ is the weight-average molecular weight (kDa) of fraction i.

(4) Fourier-Transform Infrared (FTIR) Spectroscopy

Briefly, 1–2 mg of dried polysaccharide sample was mixed with 200 mg of KBr powder and ground thoroughly in an agate mortar before pellet pressing. FTIR spectra were acquired using a Fourier-transform infrared spectrometer (Nicolet 6700, Thermo Fisher Scientific, Waltham, MA, USA) over the wavenumber range of 4000–400 cm^−1^.

### 2.8. SMP Antioxidant Activity Assays

(1) Scavenging Capacity of DPPH

The procedure was slightly modified based on a previously reported method [18]. Aliquots of 2.0 mL sample solutions at concentrations of 0.1, 0.5, 1.0, 1.5 and 2.0 mg/mL were mixed with 2.0 mL of DPPH solution (0.1 mmol/L, prepared in 75% ethanol). After thorough mixing, the mixtures were incubated at room temperature in the dark for 30 min, and the absorbance was measured at 517 nm. Vitamin C (VC) was used as the positive control. All assays were carried out in triplicate. The DPPH radical scavenging activity was calculated according to Equation (5):

(5) $$DPPH\;scavenging\;rate\;(\%)=(1-\frac{A1\;-\;A2}{A0})\times 100\%$$

where: A1—absorbance of sample solution mixed with DPPH-75% ethanol solution; A2—absorbance of sample solution mixed with 75% ethanol solution; A0—absorbance of DPPH-75% ethanol solution mixed with 75% ethanol solution.

(2) Scavenging Capacity of ABTS^+^

The procedure was slightly modified based on a previously reported method [19]. First, 25.0 mL of ABTS solution (7.0 mmol/L) was mixed with 440.0 µL of ammonium persulfate solution (140.0 mmol/L), and the mixture was incubated in the dark for 12–16 h to prepare the stock ABTS solution. Prior to use, the stock ABTS solution was diluted with 70% ethanol to obtain the working solution with an absorbance of 0.7. Subsequently, 0.8 mL sample solutions at concentrations of 0.1, 0.5, 1.0, 1.5, and 2.0 mg/mL were mixed with 3.2 mL of ABTS working solution. After mixing, the reaction system was kept in the dark at room temperature for 6 min, and the absorbance was measured at 734 nm. VC was used as the positive control. Each treatment was performed in triplicate. The ABTS radical scavenging activity was calculated according to Equation (6):

(6) $${ABTS}^{+}\;scavenging\;rate\;(\%)=\frac{A0\;-\;(A1\;-\;A2)}{A0}\times 100\%$$

where: A1—absorbance of sample solution mixed with ABTS working solution; A2—absorbance of sample solution mixed with distilled water; A0—absorbance of distilled water mixed with ABTS working solution.

(3) Scavenging Capacity of ·OH

The procedure was slightly modified based on a previously reported method [20]. First, 200.0 μL sample solutions at concentrations of 6.0, 7.0, 8.0, 9.0 and 10.0 mg/mL were mixed with 200.0 μL of ferrous sulfate solution (6.0 mmol/L) and 200.0 μL of salicylic acid–ethanol solution (6.0 mmol/L). Subsequently, 200.0 μL of 0.1% H_2_O_2_ solution was added to initiate the reaction. The mixture was incubated at 37 °C for 30 min, and the absorbance was measured at 510 nm. VC was used as the positive control. All assays were performed in triplicate. The hydroxyl radical scavenging activity was calculated according to Equation (7):

(7) $$\cdot OH\;scavenging\;rate\;(\%)=(1-\frac{A1\;-\;A2}{A0})\times 100\%$$

where: A1—absorbance of the mixture containing the sample solution, FeSO_4_ solution, salicylic acid–ethanol solution and H_2_O_2_ solution; A2—absorbance of the mixture containing the sample solution, FeSO_4_ solution, salicylic acid–ethanol solution and H_2_O; A0—absorbance of the mixture containing H_2_O, FeSO_4_ solution, salicylic acid–ethanol solution and H_2_O_2_ solution.

(4) Total reducing power

The procedure was slightly modified based on a previously reported method [21]. Aliquots of 0.8 mL sample solutions at concentrations of 0.1, 0.5, 1.0, 1.5, and 2.0 mg/mL were mixed with 1.0 mL of phosphate buffer (PB, pH 6.6) and 1.0 mL of 1% potassium ferricyanide solution. The mixture was incubated in a water bath at 50 °C for 20 min. After cooling, 1.0 mL of 10% trichloroacetic acid (TCA) was added, followed by centrifugation at 3000 r/min for 10 min. Subsequently, 2.0 mL of supernatant was collected and blended with 2.0 mL of distilled water and 0.4 mL of 1% ferric chloride hexahydrate solution. After standing for 10 min, the absorbance was measured at 700 nm. VC was used as the positive control, and all assays were performed in triplicate.

For antioxidant assays, the IC_50_ values were calculated by fitting concentration–response data to a four-parameter logistic model. When the maximal scavenging rate remained below 50% within the tested concentration range, IC_50_ was estimated by extrapolation. Such extrapolated values serve only as semi-quantitative indicators for comparing relative inhibitory activities.

### 2.9. Inhibition of α-Glucosidase and Pancreatic Lipase Activities by SMPs

(1) α-Glucosidase Inhibitory Activity of SMPs

The procedure was slightly modified based on a previously reported method [22]. Aliquots of 40.0 μL of α-glucosidase solution (1 U/mL) were mixed with 40.0 μL sample solutions at concentrations of 30.0, 60.0, 90.0, 120.0 and 150.0 μg/mL. The mixture was pre-incubated at 37 °C for 10 min, followed by the addition of 20.0 μL of PNPG solution (5.0 mmol/L). After gentle mixing, the system was incubated at 37 °C for 30 min. Subsequently, 100.0 μL of 0.1 mol/L Na_2_CO_3_ solution was added to terminate the catalytic reaction. After thorough mixing, the absorbance was measured at 405 nm, and the whole reaction process was protected from light. Acarbose was used as the positive control, and all assays were performed in triplicate. The α-glucosidase inhibition rate of the samples was calculated according to Equation (8). The inhibition rates of α-glucosidase at different polysaccharide concentrations were imported into GraphPad Prism 10.1.2 (GraphPad Software, San Diego, CA, USA). The concentration–inhibition curve was fitted by a four-parameter logistic model to calculate the IC_50_ values of each sample. All data were expressed as mean ± standard deviation.

(8) $$\alpha \text{-}glucosidase\;activity\;inhibition\;(\%)=\frac{A2\;-\;A1}{A2\;-\;A0}\times 100\%$$

where: A1—absorbance of the mixture containing α-glucosidase, sample solution, PNPG solution and Na_2_CO_3_ solution; A2—absorbance of the mixture containing a-glucosidase, PBS, PNPG solution and Na_2_CO_3_ solution; A0—absorbance of the mixture containing PBS, PBS, PNPG solution and Na_2_CO_3_ solution.

(2) Pancreatic Lipase Inhibitory Activity of SMPs

The procedure was slightly modified based on a previously reported method [23]. Aliquots of 50.0 μL sample solutions at concentrations of 2.0, 4.0, 6.0, 8.0 and 10.0 mg/mL were mixed with 50.0 μL of pancreatic lipase solution (4.0 mg/mL). After incubation at 37 °C for 20 min, 100.0 μL of PNPB solution (2.0 mmol/L) was added and mixed thoroughly. The mixture was incubated at 37 °C for another 15 min, followed by a boiling-water bath treatment for 5 min to terminate the reaction. The absorbance was measured at 405 nm, and the whole reaction process was protected from light. Orlistat was used as the positive control. Each treatment was performed in triplicate. The pancreatic lipase inhibition rate of the samples was calculated according to Equation (9). The inhibition rates corresponding to different polysaccharide concentrations were imported into GraphPad Prism 10.1.2 software. The concentration–inhibition curve was fitted using a four-parameter logistic model to calculate the IC_50_ values of each sample. All data were expressed as mean ± standard deviation.

(9) $$pancreatic\;lipase\;activity\;inhibition\;(\%)=(1\;-\;\frac{A1\;-\;A2}{A3\;-\;A4})\times 100\%$$

where: A1—absorbance of the mixture containing the sample solution, pancreatic lipase and PNPB solution; A2—absorbance of the mixture containing the sample solution, Tris-HCI buffer and PNPB solution; A3—absorbance of the mixture containing Tris-HCI buffer, pancreatic lipase and PNPB solution; A4—absorbance of the mixture containing Tris-HCI buffer and PNPB solution.

### 2.10. In Vitro Simulated Gastrointestinal Digestion of SMPs

Referring to the method described by Yu [24] with slight modifications, four kinds of SMPs (SD-SMP, HAD-SMP, WMHAD-SMP and VFD-SMP) were subjected to in vitro digestion treatment. Four sampling time points were set: before digestion, 2 h of gastric digestion, 2 h of intestinal digestion, and 4 h of intestinal digestion. The release rate of total polysaccharides, as well as the changes in DPPH radical scavenging activity, α-glucosidase inhibitory activity and pancreatic lipase inhibitory activity, were determined. Simulated gastric fluid (pH 1.2, containing pepsin) and simulated intestinal fluid (pH 6.8, containing pancreatin and bile salts) were prepared and preheated at 37 °C for subsequent use. Polysaccharide stock solutions (2.0 mg/mL) were prepared individually. An equal volume of simulated gastric fluid was mixed with each polysaccharide stock solution to achieve a final polysaccharide concentration of 1.0 mg/mL in the reaction system. Aliquots were collected immediately, centrifuged at 8000 r/min for 10 min, and filtered through a 0.22 μm membrane to obtain pre-digestion samples. The mixture was incubated at 37 °C with shaking at 120 r/min under light protection for 2 h. Samples were harvested and filtered to obtain samples after 2 h gastric digestion. The pH of the remaining mixture was adjusted to 6.8 using 0.1 mol/L NaOH solution, followed by the addition of an equal volume of preheated simulated intestinal fluid for further incubation. Samples were collected and filtered at 2 h and 4 h of cumulative intestinal digestion, respectively. The actual total polysaccharide concentration of all supernatants was determined first. Subsequently, each supernatant was normalized and diluted to a unified polysaccharide concentration before the detection of various indicators. For the DPPH radical scavenging assay and pancreatic lipase inhibitory assay, the polysaccharide concentration was adjusted to 2.0 mg/mL; for the α-glucosidase inhibitory assay, the samples were diluted to 50 μg/mL polysaccharide to eliminate non-differentiable data caused by saturation at high concentrations.

The total polysaccharide content was quantified by the phenol–sulfuric acid method, and reducing sugar content was determined using the 3,5-dinitrosalicylic acid (DNS) colorimetric method. The hydrolysis degree of SMPs was calculated accordingly, and the calculation formula for the polysaccharide hydrolysis degree was as follows:

(10) $$Hydrolysis\;degree=\frac{\mathrm{Released}\;\mathrm{reducing}\;\mathrm{sugar}\;}{\mathrm{Total}\;\mathrm{sugar}\;-\;\mathrm{initial}\;\mathrm{reducing}\;\mathrm{sugar}}\times 100\%$$

Released reducing sugar = Reducing sugar after hydrolysis − Initial reducing sugar(11)

The measurement methods for DPPH radical scavenging activity, α-glucosidase inhibitory activity and pancreatic lipase inhibitory activity were the same as those described in Section 2.8 and Section 2.9 of this article.

### 2.11. Statistical Analysis

All experiments were performed with three independent extraction replicates (biological replicates). Each independent extract was further analyzed in triplicate for analytical precision. Data are expressed as mean ± standard deviation of the independent extraction replicates (*n* = 3). Statistical analyses were conducted using SPSS 26.0 software (IBM Corporation, Armonk, New York, NY, USA). One-way analysis of variance (ANOVA) combined with Duncan’s multiple range test was used for statistical evaluation. Values of *p* < 0.05 were considered statistically significant.

## 3. Results

### 3.1. Effects of Different Drying Methods on the Physicochemical Quality of Seed Melon Dried Products

Heat and mass transfer behaviors during fruit and vegetable drying directly alter the moisture content, rehydration capacity and color attributes of dried products, which serve as intuitive indicators for evaluating drying performance [25]. Different drying methods exerted varying effects on the moisture content, rehydration characteristics and color quality of seed melon slices, as summarized in Table 1. As shown in Table 1, both SD and WMHAD reduced the moisture content of dried seed melon to below 4%, which was significantly lower than those of the HAD (9.97 ± 0.2%) and VFD (10.23 ± 0.2%) groups. This indicated that natural sun-drying and the coupled weak microwave–hot-air drying strategy could achieve deep dehydration of fruit and vegetable tissues. Nevertheless, SD required an extremely long drying duration. As illustrated in Figure 1, severe tissue shrinkage and pore collapse were observed in SD samples. The SD samples exhibited the lowest lightness value (L* = 62.57 ± 1.3) and rehydration ratio (309.52 ± 9.9%), together with a high yellowness value (b* = 26.81 ± 0.3), leading to a dull appearance and inferior overall quality. Compared with the SD group, the WMHAD samples possessed improved color and rehydration properties with remarkably suppressed color deterioration. Benefiting from the enhanced internal mass transfer induced by microwave irradiation, WMHAD samples exhibited a more favorable porous microstructure than SD samples.

The HAD samples suffered obvious deterioration in visual quality, with the highest redness (a* = 7.22 ± 1.3), yellowness (b* = 32.79 ± 1.0) and total color difference (ΔE = 21.51 ± 1.1) among all treatments, revealing severe browning. Their rehydration ratio reached 467.41 ± 11.7%, only slightly lower than that of the VFD group. VFD samples displayed the optimal color performance, with the highest L* value of 76.62 ± 1.7, a low a* value of 1.41 ± 0.4 and the minimum ΔE of 4.15 ± 0.8, presenting bright and uniform flesh color. In addition, the VFD group achieved the maximum rehydration ratio of 476.1 ± 12.3%. However, the moisture content of freeze-dried samples reached 10.23 ± 0.2%, which was markedly higher than that of the other three groups.

### 3.2. Yield and Chemical Composition Content of SMPs

The four drying methods exerted significant effects on the polysaccharide yield, total sugar, uronic acid, and protein contents. The tissue structure of raw materials directly affects the dissolution efficiency and chemical composition of polysaccharides [26]. Obvious gradient differences in the yields of SMPs were observed among the four drying treatments. As determined, the yields of crude polysaccharides extracted from seed melon subjected to SD, HAD, WMHAD and VFD were 9.8%, 12.1%, 15.5% and 18.9%, respectively.

The total carbohydrate content of SMP samples was determined to quantitatively characterize the purity of the dominant carbohydrate fraction in polysaccharides, and to compare the retention and enrichment of core saccharide substances in SMPs affected by different drying pretreatment processes [26]. As presented in Table 2, VFD-SMP (87.50 ± 0.28%) and WMHAD-SMP (87.45 ± 1.62%) had the highest total carbohydrate contents, with no significant difference between them (*p* > 0.05). Both values were significantly higher than those of HAD-SMP (84.50 ± 0.56%) and SD-SMP (80.80 ± 0.84%) (*p* < 0.05), demonstrating that VFD and WMHAD treatments exhibited favorable retention efficiency for the active constituents of SMPs. Uronic acid serves as the characteristic structural unit of acidic polysaccharides. Its content can reflect the effects of drying methods on the structural fractions of acidic SMPs and provide a theoretical basis at the structural component level for subsequent differences in physicochemical properties and antioxidant activities of SMPs [27]. The uronic acid content followed the order VFD-SMP (10.22 ± 1.04%) > HAD-SMP (8.82 ± 0.54%) ≈ SD-SMP (8.37 ± 0.29%) ≈ WMHAD-SMP (8.36 ± 1.24%). This result indicates that VFD can preserve the carboxylic acidic groups on polysaccharide molecules to the maximum extent, whereas HAD, SD and WMHAD lead to varying degrees of degradation and loss of acidic saccharide units. Residual protein in polysaccharide samples was determined to evaluate the impurity removal efficiency of the whole procedure including decolorization, deproteinization and dialysis [28]. As shown in Table 2, the residual protein levels of all samples were generally low. WMHAD-SMP possessed the lowest protein content of only 0.22 ± 0.05%, which was significantly lower than SD-SMP (0.29 ± 0.03%) and HAD-SMP (0.32 ± 0.02%). This result suggests that weaker binding interaction occurs between polysaccharides and proteins in WMHAD-treated pulp, achieving superior purification and impurity removal performance.

### 3.3. Characterization of SMPs Dried by Different Methods

SEM was used to observe the micromorphology of polysaccharides, which could visually reveal the aggregation state, surface wrinkles and pore compactness of polysaccharide molecules and provide microstructural evidence for the differences in polysaccharide characteristics and bioactivities induced by different drying pretreatments of raw materials [29]. The SEM images of the four groups of polysaccharides are shown in Figure 2. At a magnification of 100×, VFD-SMP exhibited an interwoven fibrous loose porous network structure without obvious shrinkage or collapse. This indicated that ice sublimation during VFD dehydration retained the polysaccharide molecular skeleton relatively intact. Both SD-SMP and HAD-SMP displayed dense irregular sheet structures. SD-SMP possessed a rough surface with torn edges, while HAD-SMP showed angular flakes with no internal pores. WMHAD-SMP suffered severe fragmentation of lamellar structures, presenting fine fragments with surface wrinkles. Under high magnification at 1000×, the freeze-dried polysaccharide showed thin and stretched lamellae, whereas the polysaccharide sheets of the other three groups stacked tightly.

Monosaccharide composition is a vital structural indicator that determines the basic backbone, physicochemical properties and functional potential of plant polysaccharides [30]. In this study, the PMP-derivatization HPLC method was adopted to analyze the monosaccharide components of SMPs obtained by four drying processes. The chromatograms and molar proportions of monosaccharides for each group are presented in Figure 3a and Table 3, respectively. Compared with the chromatogram of mixed standards, Fuc and GalN were not detected in all four SMP samples. The polysaccharide backbone was mainly composed of Gal, Ara and Glc, accompanied by small amounts of Rha, Xyl, Man, as well as GalA, GlcA and GlcN. Accordingly, these polysaccharides belong to typical heteropolysaccharides containing acidic saccharides (Figure 3a). Different drying processes exerted significant regulatory effects on the relative contents of monosaccharides (Table 3). As illustrated in Table 3, SD-SMP possessed the highest proportion of galactose among all groups (45.83%), maintaining the natural neutral sugar ratio of raw materials to the greatest extent. The molar proportions of Glc and Rha in HAD-SMP increased obviously, reaching 19.50% and 6.22%, respectively, while the GalA proportion was only 3.82%. For WMHAD-SMP, the proportion of backbone Gal remained stable at 44.52%, and branched-chain monosaccharides including Ara and Xyl slightly increased to 22.32% and 6.90%, with minor overall fluctuations in monosaccharide composition. In VFD-SMP, GalA was enriched to 19.63%, whereas the proportion of neutral backbone Gal decreased to 32.40%.

The molecular weight of polysaccharides is a critical indicator for judging whether polysaccharide degradation occurs. Molecular weight directly affects the diffusion rate of polysaccharides and serves as an essential structural parameter for evaluating biological functional potential [30]. The molecular weights of polysaccharides were determined via HPGPC. A calibration curve was established using dextran standards. The linear regression equation was LogMw = −0.00475V^3^ + 0.212V^2^ − 3.45V + 25.2, with R^2^ = 0.9998, indicating favorable linear fitting performance, which could be applied for the accurate calculation of polysaccharide molecular weight. In the equation, Mw represents weight-average molecular weight, and V denotes the elution volume (mL). The elution chromatograms and molecular weight distribution data of polysaccharides are shown in Figure 3b and Table 4, respectively. All SMPs obtained from the four drying processes exhibited multi-peak elution profiles, indicating that they belonged to a polydisperse polysaccharide mixture composed of high-, medium- and low-molecular-weight fractions. The order of average weight-average molecular weight was VFD-SMP (198.69 kDa) > SD-SMP (79.70 kDa) > WMHAD-SMP (59.15 kDa) > HAD-SMP (31.49 kDa). As illustrated in Table 4, the peak area proportion of small-molecule Peak 3 in HAD-SMP reached 44.31%, while the proportion of high-molecular-weight main Peak 1 was only 33.25%, resulting in the lowest overall average molecular weight. This reveals that severe polysaccharide degradation occurred under such drying conditions. WMHAD-SMP was dominated by medium-molecular-weight fractions (Peak 2), which occupied a peak area ratio of 55.26%, suggesting slight polysaccharide degradation. Medium- and low-molecular-weight fractions coexisted in SD-SMP. The proportion of high-molecular-weight Peak 1 in VFD-SMP was 22.54%, and its average molecular weight was significantly higher than those of the other three groups (*p* < 0.05), demonstrating that this drying method preserved intact macromolecular polysaccharide chains to the maximum extent.

FTIR spectroscopy was employed to characterize the fundamental sugar chain backbone and functional group types of polysaccharides [31]. As shown in Figure 3c, all four samples exhibited similar absorption features: a broad and strong absorption peak near 3400 cm^−1^ assigned to the stretching vibration of –OH groups in polysaccharide molecules, reflecting the intramolecular hydrogen-bond network [32]; the absorption peak at 2920 cm^−1^ originated from C–H stretching vibration of sugar rings [33]; a characteristic peak within 1630–1650 cm^−1^ corresponded to C=O stretching vibration of carboxyl groups in uronic acids, which serves as the diagnostic peak for identifying acidic polysaccharides [34]; multiple absorption bands in the fingerprint region of 1200–900 cm^−1^ were attributed to stretching vibrations of C–O and C–O–C glycosidic bonds of pyranose rings, confirming that the main chains of SMPs consisted of pyranosyl units [31]. It can be concluded that all characteristic absorption peaks of acidic heteropolysaccharides were detected in the four samples, with neither new peaks generated nor original peaks disappearing. The results demonstrated that drying treatments only changed the absorbance intensity of functional groups, without destroying the fundamental chemical backbone of SMPs. Intergroup differences in peak profiles were mainly reflected in absorbance intensity and peak position shift. The absorption intensities at 3400 cm^−1^ and 1640 cm^−1^ were higher in the VFD-SMP and WMHAD-SMPS groups. The characteristic peak intensity of the HAD-SMP group decreased obviously, accompanied by a peak shift toward higher wavenumbers. The absorbance intensity of SD-SMPS samples fell between those of the HAD-SMP and WMHAD-SMP groups.

### 3.4. Effects of Different Drying Methods on In Vitro Antioxidant Activities of SMPs

Drying treatment serves as a critical processing step affecting the molecular structure, retention of active functional groups, and biological activities of plant polysaccharides. Distinct thermal histories during drying induce remarkable variations in the molecular weight and spatial conformation of polysaccharides, thereby altering their antioxidant functions including scavenging reactive free radicals and donating reducing electrons [35].

DPPH is a stable lipophilic free radical, which is widely applied to simulate the lipid oxidation environment in food systems. The bleaching and scavenging effect of samples toward DPPH can directly evaluate their potential to quench lipophilic free radicals [36]. Within the concentration range of 0.1–1.0 mg/mL, the DPPH radical scavenging rate of HAD-SMP increased in a concentration-dependent manner and reached 82.8% at 1.0 mg/mL, after which the growth slowed down with further concentration elevation. By contrast, the scavenging rates of SD-SMP, WMHAD-SMP and VFD-SMP rose significantly from 0.1 to 1.5 mg/mL and peaked at 1.5 mg/mL with values of 77.1%, 80.6% and 90.8%, respectively (Figure 4a). According to the IC_50_ results, the DPPH scavenging IC_50_ values of SD-SMP, HAD-SMP, WMHAD-SMP and VFD-SMP were 0.63, 0.23, 0.57 and 0.48 mg/mL, respectively. The antioxidant activity followed the order HAD-SMP > VFD-SMP > WMHAD-SMP > SD-SMP. Notably, HAD-SMP exhibited a markedly lower IC_50_ than the other three groups, indicating that polysaccharides obtained by hot-air drying could efficiently quench DPPH free radicals at relatively low concentrations and possessed the optimal antioxidant capacity.

The ABTS^+^ aqueous cation radical system is suitable for detection under aqueous conditions and can comprehensively reflect the overall capacity of samples to scavenge various water-soluble reactive free radicals, serving as a universal method for evaluating the antioxidant activity of polysaccharides [37]. Within the concentration gradient of 0.1–2.0 mg/mL, the ABTS^+^ radical scavenging rates of the four polysaccharide samples continuously increased with rising concentration and reached the maximum values at 2.0 mg/mL. The peak scavenging rates of SD-SMP, HAD-SMP, WMHAD-SMP and VFD-SMP were 42.57%, 77.80%, 54.26% and 55.66%, respectively (Figure 4b). The calculated ABTS^+^ scavenging IC_50_ values of SD-SMP, HAD-SMP, WMHAD-SMP and VFD-SMP were 2.85, 0.89, 1.71 and 1.62 mg/mL, respectively. Among them, the IC50 value of SD-SMP (2.85 mg/mL) exceeded the highest tested concentration of 2.0 mg/mL and was therefore obtained by extrapolation. The activity order was completely consistent with that obtained from the DPPH assay: HAD-SMP > VFD-SMP > WMHAD-SMP > SD-SMP. These results demonstrate that HAD-SMP exhibited the optimal antioxidant activity in both DPPH and ABTS^+^ radical scavenging systems. Hot-air drying enabled SMPs to quench lipophilic free radicals more efficiently and endowed them with stronger electron-donating capacity against stable organic cation radicals.

Hydroxyl radicals (·OH) are the most destructive reactive free radicals in living organisms and food systems. They readily attack biomacromolecules such as polysaccharides and proteins and trigger oxidative degradation. This assay can evaluate the protective potential of polysaccharides against severe oxidative damage [38]. Within the concentration range of 6–10 mg/mL, the ·OH scavenging rates of SD-SMP, HAD-SMP, WMHAD-SMP and VFD-SMP increased with rising concentration, with maximum scavenging rates of 30.00%, 41.58%, 74.55% and 63.64%, respectively (Figure 4c). The IC_50_ order in the ·OH system differed from those of the previous two radical assays. The IC50 values of SD-SMP, HAD-SMP, WMHAD-SMP, and VFD-SMP in the ·OH system were 16.25, 13.06, 5.96, and 8.31 mg/mL, respectively. The IC50 values of SD-SMP and HAD-SMP exceeded the highest tested concentration (10.0 mg/mL) and were obtained by extrapolation. The antioxidant activity followed the sequence WMHAD-SMP > VFD-SMP > HAD-SMP > SD-SMP. These results indicated that SMPs prepared by different drying methods exhibited distinct scavenging capacities against various free radicals. Among them, WMHAD-SMP possessed relatively strong hydroxyl radical scavenging activity.

The total reducing power assay is based on the chromogenic reaction of ferric ion reduction. A higher absorbance value indicates a stronger capacity of samples to donate reducing electrons and interrupt oxidative chain reactions, which can directly reflect the overall reducing activity level of polysaccharide molecules [39]. As shown in Figure 4c, the reducing power of all polysaccharide samples gradually increased with the rising concentration within the range of 0.1–2.0 mg/mL. At identical concentrations, HAD-SMP exhibited remarkably stronger reducing power than the other three groups, which was consistent with the tendency of DPPH and ABTS^+^ radical scavenging activities. This further verifies that hot-air-dried polysaccharides contain more reductive functional groups and possess superior electron-donating capacity. SD-SMP displayed the lowest reducing power throughout the concentration gradient, which mutually confirmed the results from the radical scavenging assays.

### 3.5. Effects of Different Drying Methods on α-Glucosidase and Pancreatic Lipase Inhibitory Activities of SMPs

Polysaccharides can competitively inhibit key enzymes involved in carbohydrate and lipid digestion by binding to the active sites of digestive enzymes and shielding substrate-binding regions. A lower IC_50_ value of inhibition rate corresponds to stronger inhibitory capacity. This serves as a classic in vitro evaluation method for assessing the hypoglycemic and lipid-lowering potential of polysaccharides [40].

α-glucosidase is the key rate-limiting enzyme responsible for breaking down starch and oligosaccharides into glucose in the small intestine. Inhibition of this enzyme can retard postprandial blood glucose elevation, rendering it a core in vitro model to evaluate the hypoglycemic potential of polysaccharides [41]. Within the concentration range of 30–150 μg/mL, the α-glucosidase-inhibitory rates of the four SMPs increased significantly as the sample concentration rose, showing a typical dose-dependent effect (Figure 5a). The α-glucosidase inhibitory IC50 values (μg/mL) of VFD-SMP, WMHAD-SMP, SD-SMP, and HAD-SMP were 71.21 ± 3.11, 85.29 ± 3.66, 97.02 ± 4.49, and 135.43 ± 5.50, respectively. The inhibitory activity followed the order VFD-SMP > WMHAD-SMP ≈ SD-SMP > HAD-SMP.

Pancreatic lipase catalyzes the hydrolysis of dietary triglycerides in the intestinal tract. Inhibition of its activity reduces the absorption of fatty acids, and this assay serves as a standard in vitro system for evaluating the lipid-lowering activity of natural polysaccharides [42]. Within the tested concentration range of 2.0–10.0 mg/mL, the pancreatic lipase inhibitory rates of the four polysaccharides increased continuously with rising concentration, showing an obvious dose–response effect (Figure 5c). The IC_50_ values (mg/mL) of WMHAD-SMP, VFD-SMP, SD-SMP, and HAD-SMP against pancreatic lipase were 3.13 ± 0.39, 3.60 ± 0.24, 5.65 ± 0.56, and 9.77 ± 0.64, respectively. The inhibitory activity followed the order WMHAD-SMP ≈ VFD-SMP > SD-SMP > HAD-SMP.

### 3.6. Effects of Different Drying Methods on Gastrointestinal Digestion Stability of SMPs

The molecular retention rate and activity attenuation of polysaccharides during gastrointestinal digestion serve as important indicators for evaluating the industrial application prospects of polysaccharides [41].

The hydrolysis degree of four types of SMPs increased significantly with the prolongation of simulated gastrointestinal digestion, and the hydrolysis efficiency exhibited obvious dependence on digestion stages. At the stage of 2 h gastric digestion, all polysaccharide samples showed relatively low hydrolysis levels. After transferring to the small-intestinal digestion stage, the altered digestive environment remarkably accelerated the enzymatic hydrolysis of polysaccharides. The hydrolysis degree of all samples reached the maximum value at 4 h of small-intestinal digestion. At each identical digestion time point, the ranking of hydrolysis degree consistently followed the order HAD-SMP > WMHAD-SMP > SD-SMP > VFD-SMP. HAD-SMP possessed the highest hydrolysis degree, while VFD-SMP exhibited the lowest hydrolysis degree after 4 h of small-intestinal digestion (Figure 6a). Generally, digestive enzymes in the simulated gastrointestinal fluid show limited capacity to degrade non-starch polysaccharides thoroughly. The superior hydrolysis performance of HAD-SMP was closely related to its molecular structure and molecular weight characteristics. Thermal degradation occurred during high-temperature hot-air drying, generating abundant low-molecular-weight fragments in HAD-SMP. These fragments were more susceptible to the cleavage of glycosidic bonds by glycosidases in digestive juice, leading to a higher hydrolysis degree.

The DPPH radical scavenging capacity of all polysaccharides increased continuously with extended digestion time. A remarkable enhancement was observed after 2 h of gastric digestion, whereas the growth rate slowed down and gradually plateaued during intestinal digestion. The activity ranking remained consistent throughout the whole digestion period: HAD-SMP > VFD-SMP > WMHAD-SMP > SD-SMP. Undigested HAD-SMP exhibited the best intrinsic antioxidant activity. During digestion, HAD-SMP underwent the highest degree of degradation and generated the largest amount of low-molecular-weight reductive products, resulting in a significantly greater increase in activity compared with the other three groups. Although VFD-SMP displayed the slowest degradation rate, its initial antioxidant activity was second only to HAD-SMP, enabling it to maintain a relatively high scavenging level over the entire digestion process (Figure 6b).

The α-glucosidase inhibitory rates of the four polysaccharides declined continuously as digestion proceeded. The inhibitory activity decreased markedly after gastric hydrolysis disrupted long-chain structures, and continuous degradation in the intestinal phase further weakened the inhibitory effect, with all groups reaching the lowest inhibitory activity at 4 h of intestinal digestion. VFD-SMP exhibited the slowest decline in enzyme inhibitory activity during digestion, suggesting that its active structures may be more resistant to digestive degradation. However, because post-digestion molecular weight was not measured, the inference that intact long-chain polysaccharides were retained should be regarded as tentative and requires further verification. By contrast, HAD-SMP possessed the lowest molecular weight and underwent the highest degree of hydrolysis. Massive fragmentation of long-chain structures led to the most remarkable attenuation of its hypoglycemic inhibitory activity (Figure 6c).

The variation trend of pancreatic lipase inhibitory activity was completely consistent with that of α-glucosidase; the inhibitory effect weakened with prolonged digestion time. Intact long polysaccharide chains constitute the critical structure responsible for lipid-lowering inhibitory effects, whereas hydrolyzed small oligosaccharides and monosaccharides exhibit almost no lipase-blocking capacity. Native WMHAD-SMP possessed the optimal lipid-lowering activity. VFD-SMP retained relatively high activity after digestion, possibly due to the structural stability of its high-molecular-weight fractions. Only weak inhibitory activity was detected for HAD-SMP upon completion of gastrointestinal digestion (Figure 6d).

## 4. Discussion

Waste seed melon pulp by-products have a high moisture content and are highly susceptible to spoilage after harvest. Drying serves as a critical pretreatment to extend their shelf life and facilitate subsequent polysaccharide extraction. Temperature, oxygen exposure and mass transfer conditions during drying alter the tissue morphology of pulp, thereby affecting the molecular structure and biological functions of the extracted polysaccharides [43]. Most existing studies on melon polysaccharides focus on the optimization of extraction procedures or the preparation and bioactivity evaluation of polysaccharides obtained using only a single drying method. Research that simultaneously compares four drying methods, namely SD, HAD, WMHAD and VFD, and systematically investigates dried product quality, multi-level structures of polysaccharides, multi-target in vitro bioactivities and gastrointestinal digestive stability remains limited. To fill this research gap, the physicochemical properties, polysaccharide yield and chemical composition of dried products prepared from waste seed melon pulp by-products using different drying methods were determined. The monosaccharide composition, molecular weight distribution, micromorphology and FTIR spectra of polysaccharides were characterized, and their antioxidant activity, inhibitory activity against α-glucosidase and pancreatic lipase, as well as in vitro gastrointestinal digestive stability were evaluated, so as to elucidate the intrinsic relationship between drying-induced structural variations and functional properties of polysaccharides.

Significant differences in moisture content, color parameters and rehydration properties were observed among dried seed melon products obtained by different drying methods (Table 1). SD exhibited a slow dehydration rate under aerobic conditions, resulting in irreversible shrinkage of pulp cells and severe collapse of pore structures, accompanied by polyphenol oxidase-mediated browning, which was consistent with the browning characteristics of open-air-dried fruits and vegetables [44]. Continuous high temperature during HAD triggered Maillard and caramelization reactions and caused obvious color deterioration. Nevertheless, only partial collapse of the pore framework occurred, and favorable rehydration performance was retained, which agreed with previous reports on structure–quality relationships [45]. WMHAD combines the synergistic effects of volumetric microwave heating and mild hot-air mass transfer. It prevents materials from enduring prolonged intense thermal stress, effectively alleviates tissue structural damage and inhibits heat-induced browning reactions. Consequently, WMHAD samples showed superior color stability and lower moisture content compared with the SD and HAD groups, which were beneficial for product storage [46]. VFD achieves dehydration via ice sublimation without tissue collapse caused by liquid water migration, thus well preserving the porous structure of pulp [47]. Therefore, VFD samples possessed the best color appearance and the strongest rehydration capacity. However, this technique only removes free water, and residual bound water leads to a relatively high equilibrium moisture content in finished products, requiring sealed and moisture-proof storage, which is a typical feature of freeze-dried fruits and vegetables [48].

Differences in pulp micromorphology induced by various drying methods further affect the extraction efficiency and basic composition of polysaccharides. The crude polysaccharide yield of waste seed melon pulp by-products treated by VFD was higher than those of the other three groups, which could be attributed to the loose and porous structure of freeze-dried pulp facilitating solvent penetration and adequate polysaccharide dissolution [49]. No significant difference in total sugar content was observed between VFD and WMHAD samples, and both values were higher than those of the HAD and SD groups. This phenomenon may be explained by the continuous cleavage of polysaccharide glycosidic bonds under sustained high temperature and long-term aerobic conditions; the generated small molecular saccharides are lost during dialysis, thereby reducing the proportion of total sugars in SMPs [29]. Data in Table 2 showed that VFD-SMP possessed a significantly higher uronic acid content. The sustained thermal stress of HAD, long-term oxidation of SD, and combined effects of mild thermal action and continuous air flow in the WMHAD process may lead to the degradation of galacturonic acid structure [50]. WMHAD-SMP exhibited the lowest content of protein impurities, indicating fewer polysaccharide–protein complexes formed under this drying condition and lower difficulty in subsequent deproteinization. The above differences in basic composition imply distinctions in the molecular skeleton and reserve of active groups among SMPs from different groups. Therefore, further characterizations including monosaccharide composition, molecular weight distribution, micromorphology and FTIR spectra were performed to elucidate the regulatory effect of drying methods on the molecular structure of polysaccharides.

Structural characterization results of polysaccharides verified that external stress induced by drying could not disrupt the pyranosidic backbone of seed melon polysaccharides. Instead, structural remodeling was achieved by modulating monosaccharide ratios, degree of polymerization, micromorphology and functional group distribution, which further caused differences in biological activities [51]. Severe thermal stress during HAD promoted the depolymerization of long polysaccharide chains, leading to the loss of acidic sugar moieties and a reduction in molecular weight. Oxidation under SD slightly damaged the acidic structures on polysaccharide branches with minor destruction of the main chain. WMHAD could well retain the branched architecture of polysaccharides and increase the relative proportion of branched monosaccharides. The low-temperature and oxygen-free environment of VFD maximally protected active structural units such as galacturonic acid and maintained the integrity of acidic structures in polysaccharides. As a core structural unit, galacturonic acid may bind to digestive enzymes through electrostatic interactions, which may serve as one of the potential structural bases underlying the hypoglycemic and hypolipidemic activities of polysaccharides [52]. Differences in its content can provide indirect molecular-level evidence for the varied enzyme inhibitory capacities of different samples. Changes in molecular weight induced by drying may also affect polysaccharide bioactivities: low-molecular-weight polysaccharides generated by thermal degradation often exhibit stronger free-radical scavenging capacity, whereas high-molecular-weight acidic polysaccharides may exert enzyme barrier and inhibitory effects to a certain extent via the formation of spatial network structures [53,54]. Micromorphology and FTIR spectra further corroborated the above-mentioned potential structure–activity relationship: VFD facilitated the retention of porous structures and intact functional groups of polysaccharides; high temperature in HAD caused structural densification and collapse, accompanied by the attenuation of active functional groups; WMHAD could well maintain the overall conformation of polysaccharides [55].

In summary, drying processes synergistically modulate the molecular weight, acidic functional groups, micromorphology and branched architecture of polysaccharides, leading to differences in antioxidant capacity and digestive enzyme inhibitory activities among groups and demonstrating distinct structure–activity relationships [56,57]. In antioxidant assay systems, the homogeneous radical scavenging capacity of polysaccharides mainly relies on low-molecular-weight fragments and reductive functional groups [58]. HAD-SMP produced abundant low-molecular-weight fragments and active hydroxyl groups through thermal cleavage, showing the optimal performance in DPPH, ABTS^+^ radical scavenging and reducing power assays. Nevertheless, hydroxyl radical scavenging efficiency is largely governed by microdiffusion characteristics [59]. The fragmented microstructure of WMHAD-SMP effectively reduces diffusion resistance and compensates for its disadvantages in molecular composition. Although VFD-SMP retains intact architecture and abundant acidic groups, steric hindrance originating from high molecular weight limits its antioxidant efficacy. SD-SMP suffers prolonged oxidative damage, loss of active groups and structural densification and hardening, thus exhibiting the weakest overall antioxidant activity. In terms of enzyme inhibitory performance, the high galacturonic acid content of VFD-SMP provides abundant negatively charged carboxyl groups, which may bind to digestive enzymes via electrostatic interactions. Meanwhile, its high-molecular-weight chains may form spatial barriers, partially accounting for its strong α-glucosidase inhibitory activity. The branched architecture of WMHAD-SMP may expand molecular conformation and increase contact opportunities with pancreatic lipase, which could partly explain its prominent lipase inhibitory activity. The high-molecular-weight network structure of VFD-SMP also maintains favorable lipase inhibitory capacity. For HAD-SMP, chain scission and massive loss of acidic active moieties result in the absence of enzyme binding sites and spatial barriers, thereby yielding the weakest inhibitory effects against both digestive enzymes. SD-SMP presents moderately low overall enzyme inhibitory activity owing to impaired integrity of molecular structure and active groups.

In vitro simulated gastrointestinal digestion assays further indicated that the molecular structural characteristics of polysaccharides may be closely associated with their digestive tolerance and functional stability [60]. The short-chain structure of low-molecular-weight HAD-SMP exhibited poor structural stability and was more susceptible to hydrolysis by digestive enzymes, accompanied by the loss of active groups, resulting in a marked decline in enzyme inhibitory activity during digestion. In contrast, the dense network structure and abundant carboxyl groups of high-molecular-weight acidic VFD-SMP may endow it with strong resistance to enzymatic degradation. Thus, its active groups and inhibitory activity can be largely retained after digestion, showing relatively high gastrointestinal stability. WMHAD-SMP and SD-SMP exhibited intermediate stability. Such structure-mediated differences in stability can provide preliminary references for the application of different SMPs: VFD-SMP may be more suitable as an oral functional ingredient, whereas readily degradable HAD-SMP may be better applied to in vitro antioxidant scenarios. Nevertheless, since the molecular weight distribution of digestion products was not determined, the conclusions regarding the gastrointestinal stability of long-chain polysaccharides should be regarded as preliminary inferences.

Based on the above associations, this study proposes a potential structure–activity relationship: different drying methods can differentially modulate the antioxidant, hypoglycemic and hypolipidemic properties of SMPs by remodeling the micromorphology of waste seed melon pulp by-products and their extracted polysaccharides, thereby providing a basis for the selection of drying methods in industrial applications. Current research on the deep processing of seed melon mainly focuses on the utilization of seeds, while systematic investigations regarding the drying mechanism of pulp by-products and polysaccharide bioactivities remain limited. Moreover, most existing studies on fruit and vegetable drying merely perform a simple comparison between high-temperature and low-temperature technologies. The present work supplemented SD and WMHAD treatments to improve the comparative analysis of multiple drying systems, further enriching the theoretical foundation for the drying processing of seed melon by-products [61]. The results of process adaptability suggested that VFD appeared to better preserve the loose porous structure of pulp and the acidic structural units of polysaccharides, which may contribute to the favorable enzyme inhibitory activities and digestive stability of SMPs. Therefore, VFD-SMP has potential to be further developed as an oral functional ingredient, although its in vivo efficacy, safety and pilot-scale feasibility still need to be verified. HAD enables high-efficiency processing but may induce thermal degradation of polysaccharides. The obtained HAD-SMP exhibited prominent in vitro antioxidant activity yet limited hypoglycemic and hypolipidemic capacities, indicating its suitability for antioxidant-related applications. WMHAD avoids prolonged intense thermal stress, and the corresponding SMPs possess favorable comprehensive in vitro bioactivities, showing potential for the development of snack-type products. Although SD achieves optimal economic efficiency, it causes severe structural damage and weakens the functional bioactivities of polysaccharides, and thus may only be suitable for primary rough processing of by-products.

The present study still has certain limitations. All bioactivity evaluations were performed in in vitro simulated systems, lacking in vivo pharmacological verification. In addition, the optimization effects of combined processing technologies on polysaccharide quality were not explored, and fractionation characterization of degraded oligosaccharides was not conducted in the digestion assays. Further research can focus on in vivo activity validation, optimization of coupled processing technologies, and refined analysis of digestive products. This work provides theoretical support and data references for the high-value utilization and resource recovery of seed melon by-products in Xinjiang.

## 5. Conclusions

Four drying methods (SD, HAD, WMHAD and VFD) did not disrupt the glycosidic backbone of SMPs but differentially remodeled their monosaccharide ratios, molecular weight distribution, micromorphology and functional group profiles. VFD-SMP exhibited favorable α-glucosidase inhibition and gastrointestinal stability, HAD-SMP showed the strongest antioxidant capacity, WMHAD-SMP displayed prominent pancreatic lipase inhibition, and SD-SMP performed the worst overall. These in vitro results suggest preliminary application directions—VFD-SMP may have potential as an oral functional ingredient, HAD-SMP may be more suitable for antioxidant-related applications, and WMHAD-SMP may have potential for snack-type products—which require in vivo validation and scale-up evaluation.

## Figures and Tables

**Figure 1:**
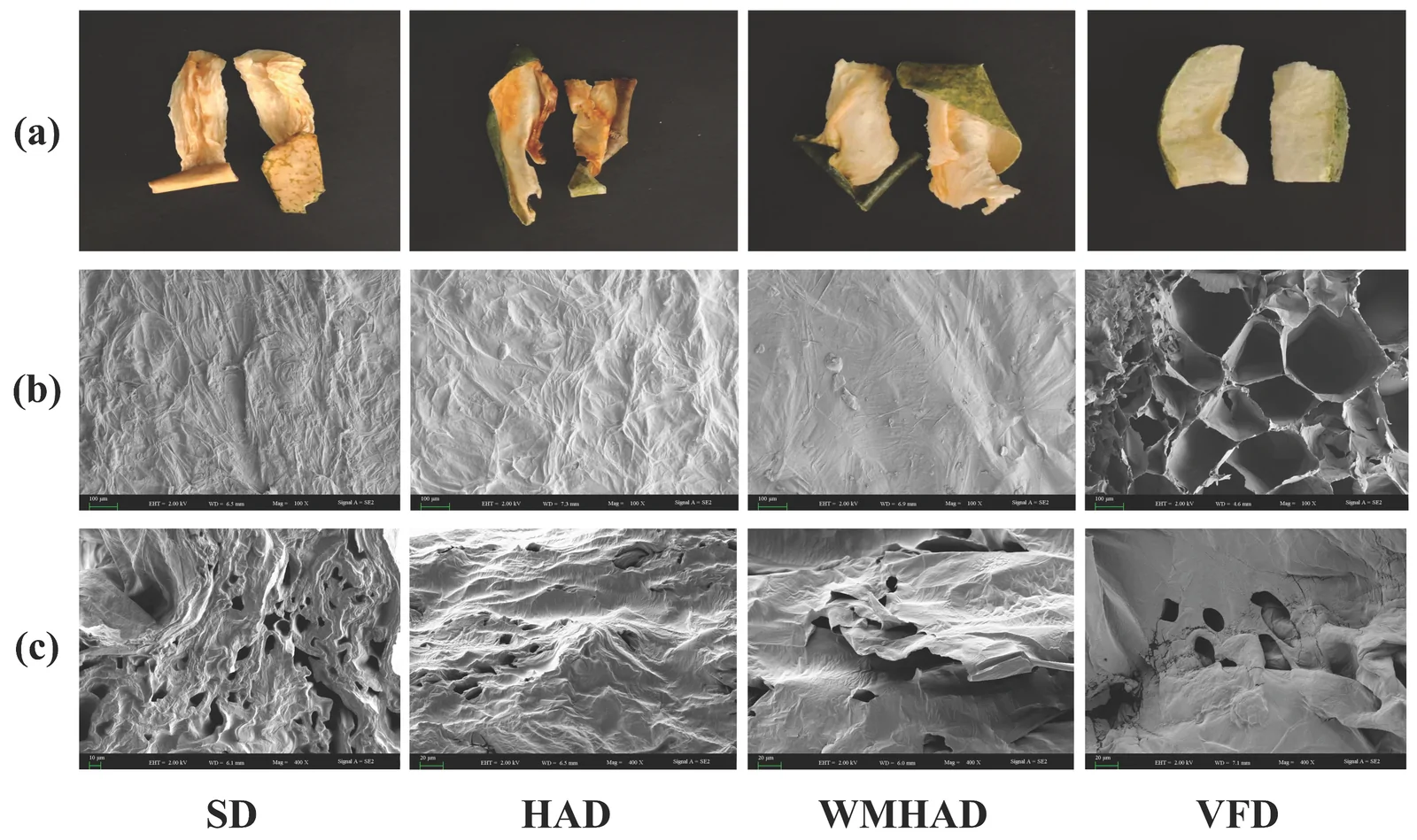
Photographs and SEM micrographs of dried seed melon prepared by different drying methods. (**a**) Photographs of all sample groups, (**b**) surface microstructure of all sample groups, and (**c**) cross-sectional microstructure of all sample groups.

**Figure 2:**
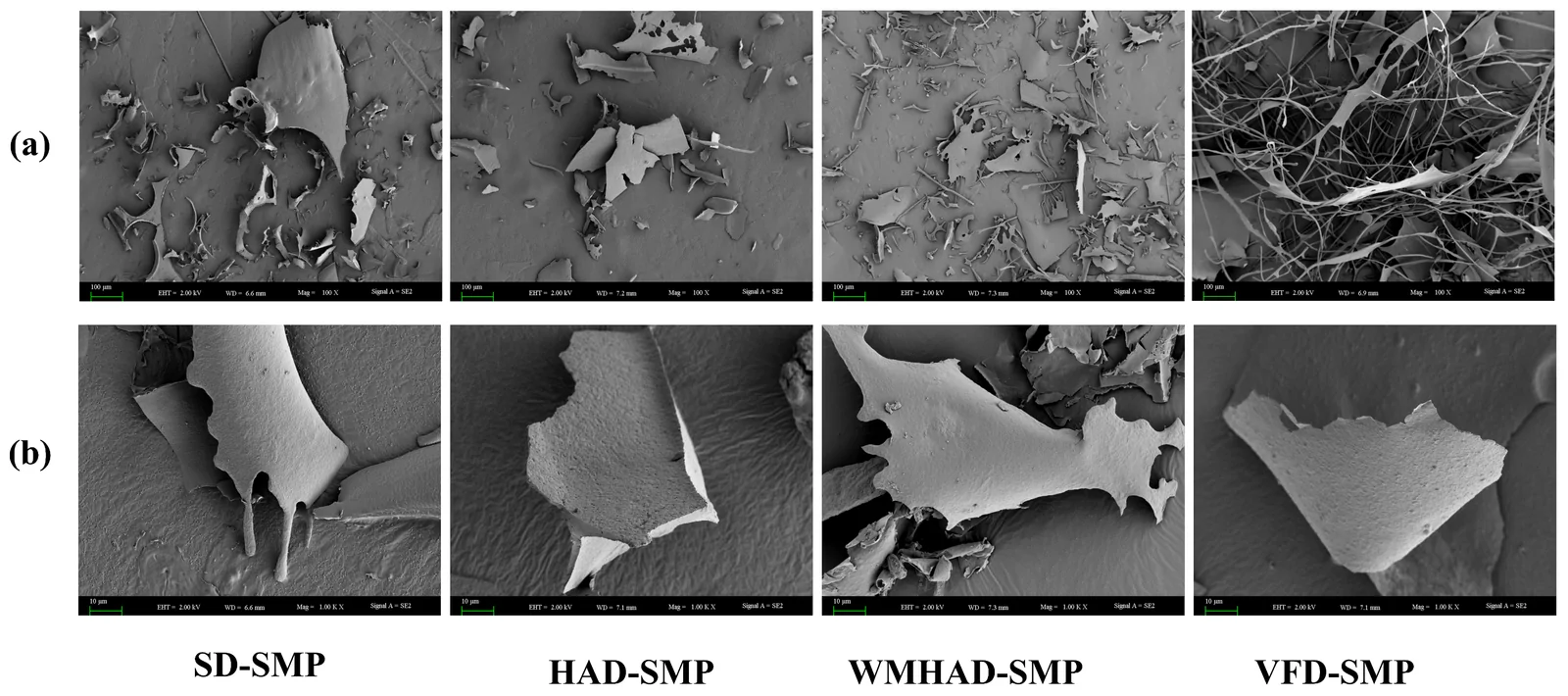
SEM micrographs of SMPs: (**a**) 100× magnification; (**b**) 1000× magnification.

**Figure 3:**
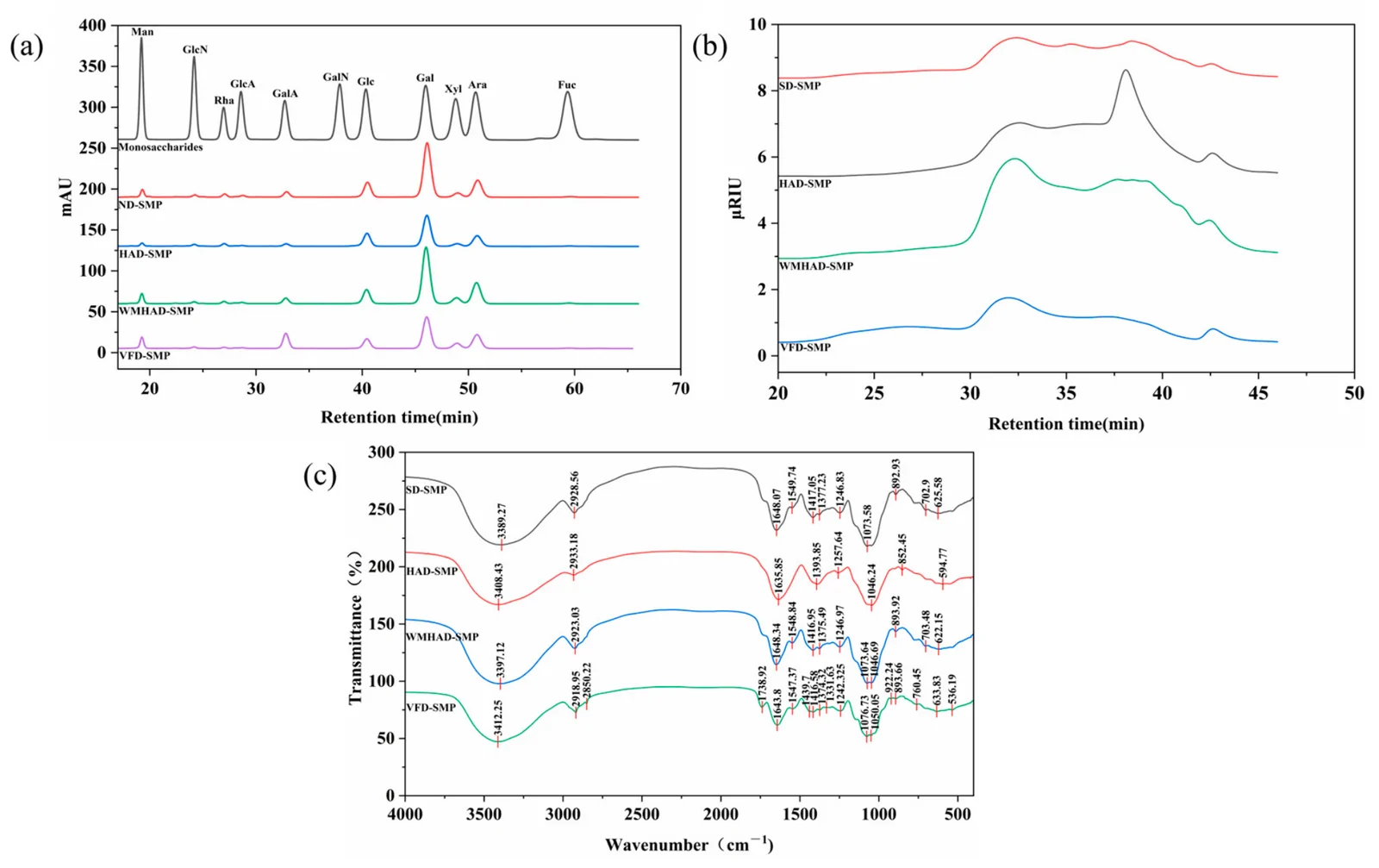
Monosaccharide composition, molecular weight distributions and FTIR characteristics of SMPs. (**a**) Monosaccharide composition; (**b**) molecular weight distribution; (**c**) FTIR spectra.

**Figure 4:**
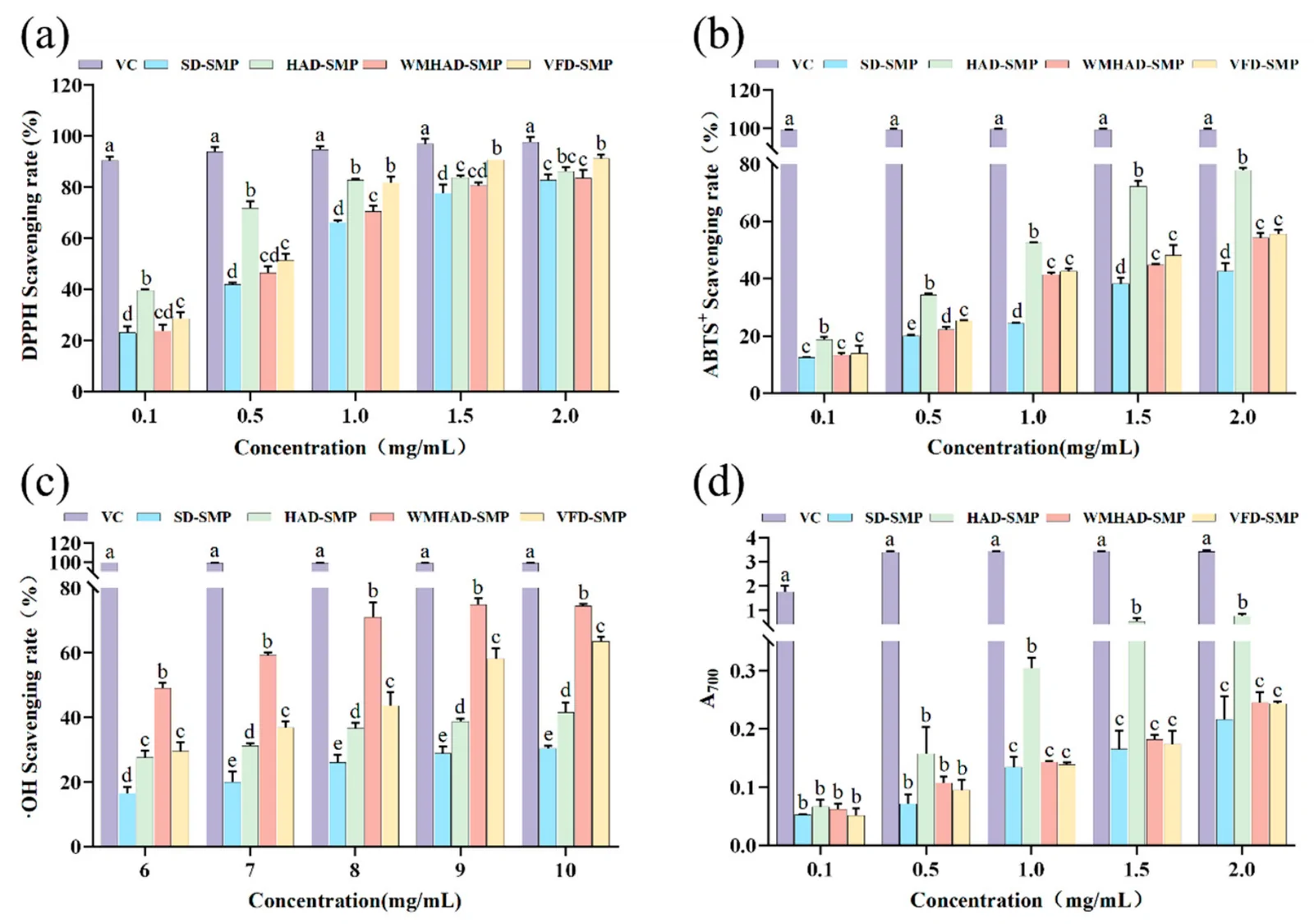
In vitro antioxidant capacities of SMPs. (**a**) DPPH scavenging rate; (**b**) ABTS^+^ scavenging rate; (**c**) ·OH scavenging rate; (**d**) total reducing power. Means in the same column with different letters are significantly different (*p* < 0.05) (Duncan’s test).

**Figure 5:**
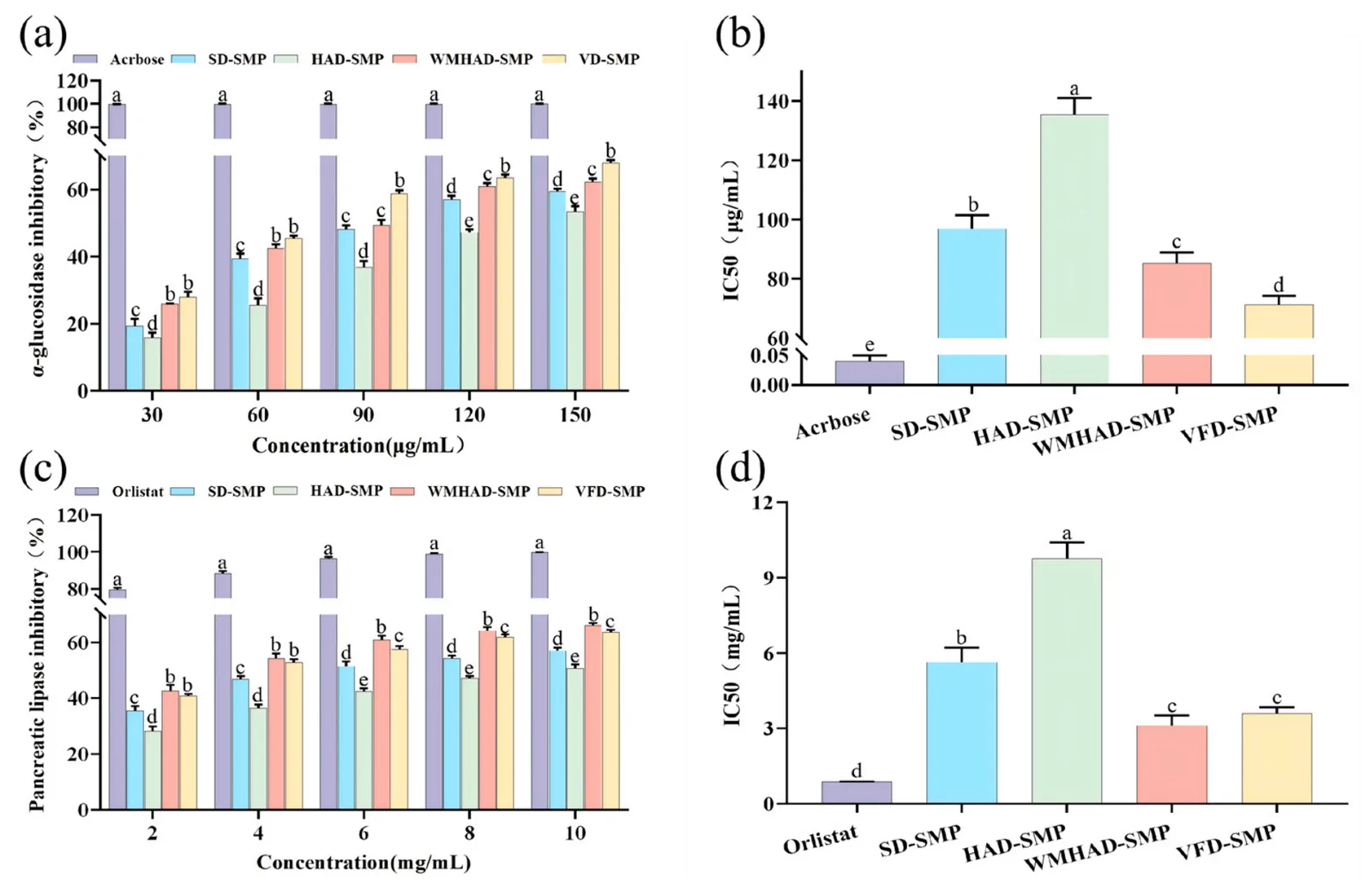
Inhibitory effects of SMPs against α-glucosidase and pancreatic lipase. (**a**) α-glucosidase inhibition; (**b**) IC_50_ values of SMPs for α-glucosidase inhibition; (**c**) pancreatic lipase inhibition; (**d**) IC_50_ values of SMPs for pancreatic lipase inhibition. Means in the same column with different letters are significantly different (*p* < 0.05) (Duncan’s test).

**Figure 6:**
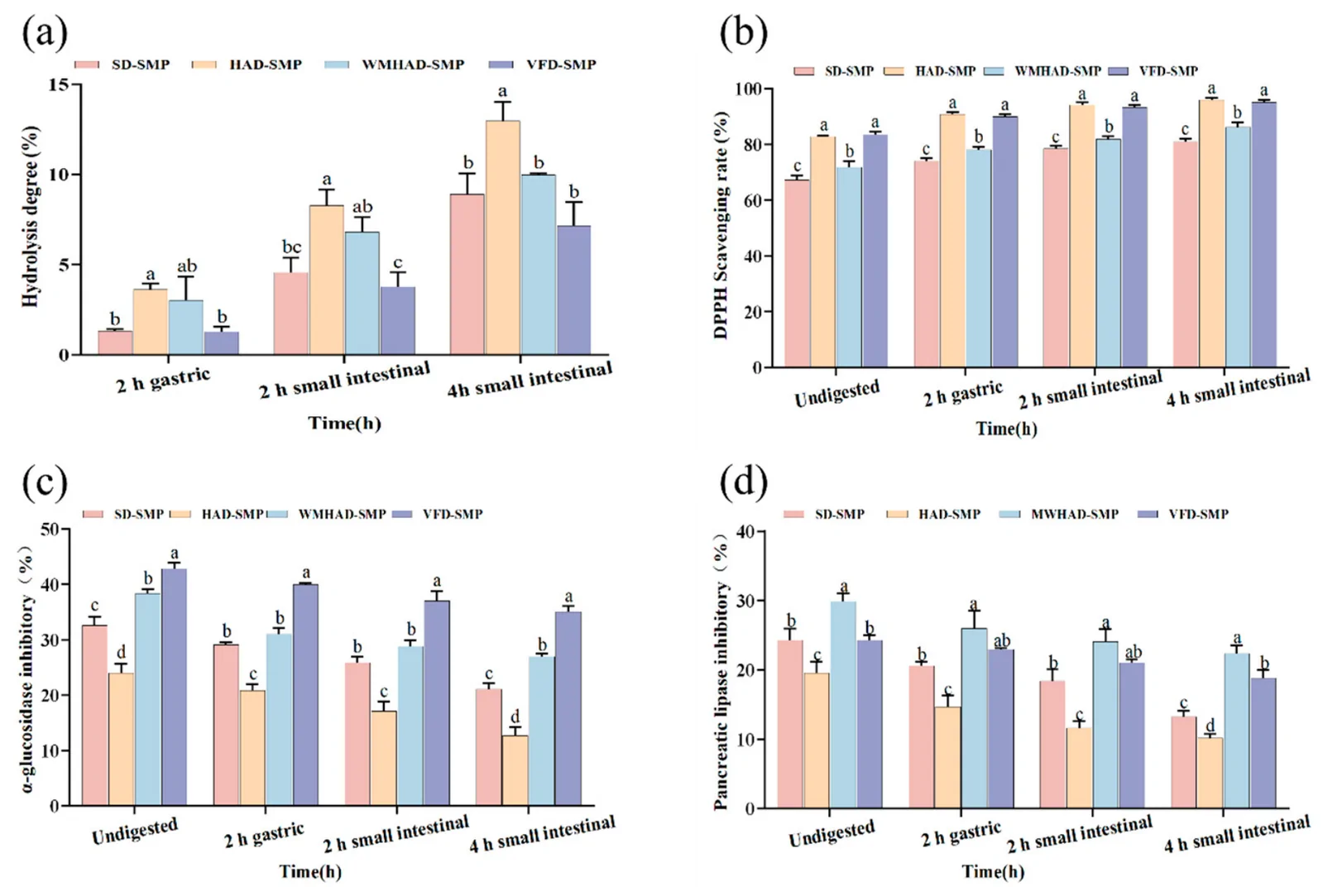
Variations in biological activities of SMPs at various gastrointestinal digestion phases. (**a**) Hydrolysis degree; (**b**) DPPH scavenging rate; (**c**) α-glucosidase inhibitory activity; (**d**) pancreatic lipase inhibitory activity. Means in the same column with different letters are significantly different (*p* < 0.05) (Duncan’s test).

**Table 1:** Moisture content, rehydration ratio, and surface color of seed melon dried by different drying methods.

Samples	Moisture Content	Rehydration Ratio	Surface Color			
			L*	a*	b*	ΔE
SD	3.98 ± 0.1 ^b^	309.52 ± 9.9 ^c^	62.57 ± 1.3 ^c^	6.56 ± 0.7 ^a^	26.81 ± 0.3 ^b^	16.54 ± 1.0 ^b^
HAD	9.97 ± 0.2 ^a^	467.41 ± 11.7 ^a^	61.50 ± 1.1 ^c^	7.22 ± 1.3 ^a^	32.79 ± 1.0 ^a^	21.51 ± 1.1 ^a^
WMHAD	3.94 ± 0.1 ^b^	342.6 ± 10.6 ^b^	72.33 ± 2.1 ^b^	7.37 ± 0.8 ^a^	26.70 ± 1.2 ^b^	11.82 ± 1.1 ^d^
VFD	10.23 ± 0.2 ^a^	476.1 ± 12.3 ^a^	76.62 ± 1.7 ^a^	1.41 ± 0.4 ^b^	20.51 ± 0.2 ^c^	4.15 ± 0.8 ^d^

Note: Means in the same column with different letters are significantly different (*p* < 0.05) (Duncan’s test), and this notation is consistent in subsequent tables.

**Table 2:** Chemical substance contents of SMPs.

Samples	Carbohydrate/%	Uronic Acid/-	Protein/%
SD-SMP	80.80 ± 0.84 ^c^	8.37 ± 0.29 ^b^	0.29 ± 0.03 ^a^
HAD-SMP	84.50 ± 0.56 ^b^	8.82 ± 0.54 ^ab^	0.32 ± 0.02 ^a^
WMHAD-SMP	87.45 ± 1.62 ^a^	8.36 ± 1.24 ^b^	0.22 ± 0.05 ^b^
VFD-SMP	87.50 ± 0.28 ^a^	10.22 ± 1.04 ^a^	0.27 ± 0.03 ^ab^

Note: Means in the same column with different letters are significantly different (*p* < 0.05) (Duncan’s test), and this notation is consistent in subsequent tables.

**Table 3:** Molar percentages of individual monosaccharides in SMPs (%).

Samples	SD-SMP	HAD-SMP	WMHAD-SMP	VFD-SMP
Rha	4.58	6.22	2.80	1.74
Ara	19.17	18.90	22.32	19.04
Gal	45.83	41.30	44.52	32.40
Glc	13.57	19.50	11.85	10.71
Xyl	5.04	4.87	6.90	7.99
Man	3.65	2.33	4.26	6.59
GalA	5.48	3.82	5.32	19.63
GlcA	1.36	0.88	0.94	0.62
GlcN	1.32	2.18	1.11	1.29

**Table 4:** Molecular weight distributions of SMPs.

Samples	SD-SMP	HAD-SMP	WMHAD-SMP	VFD-SMP
Peak 1	1509.68	81.24	1832.09	753.57
Peak area/%	1.87	33.25	0.82	22.54
Peak 2	477.59	14.63	77.28	53.45
Peak area/%	4.51	22.44	55.26	51.94
Peak 3	66.75	2.70	6.16	4.20
Peak area/%	39.84	44.31	17.91	25.52
Peak 4	13.72	/	1.23	/
Peak area/%	17.68	/	26.02	/
Peak 5	2.52	/	/	/
Peak area/%	36.11	/	/	/
Weight-average molecular weight/kDa	79.70	31.49	59.15	198.69

## Data Availability

The original contributions presented in this study are included in the article. Further inquiries can be directed to the corresponding author.

## Abbreviations

The following abbreviations are used in this manuscript:

SD	Sun drying
VFD	Vacuum freeze drying
HAD	Hot-air drying
WMHAD	Weak microwave-assisted hot-air drying
SMPs	Seed melon polysaccharides
SEM	Scanning electron microscopy
FTIR	Fourier-transform infrared
VC	Vitamin C
